# Supplementary material for: Interdependence patterns of multifrequency oscillations predict visuomotor behavior
Source: Netw Neurosci. 2025 May 8;9(2):712–42. doi: 10.1162/netn_a_00440 (PMC12140573; doi:10.1162/netn_a_00440)
Supplement: Supplementary file 1 [file netn-9-2-712-s001.pdf]

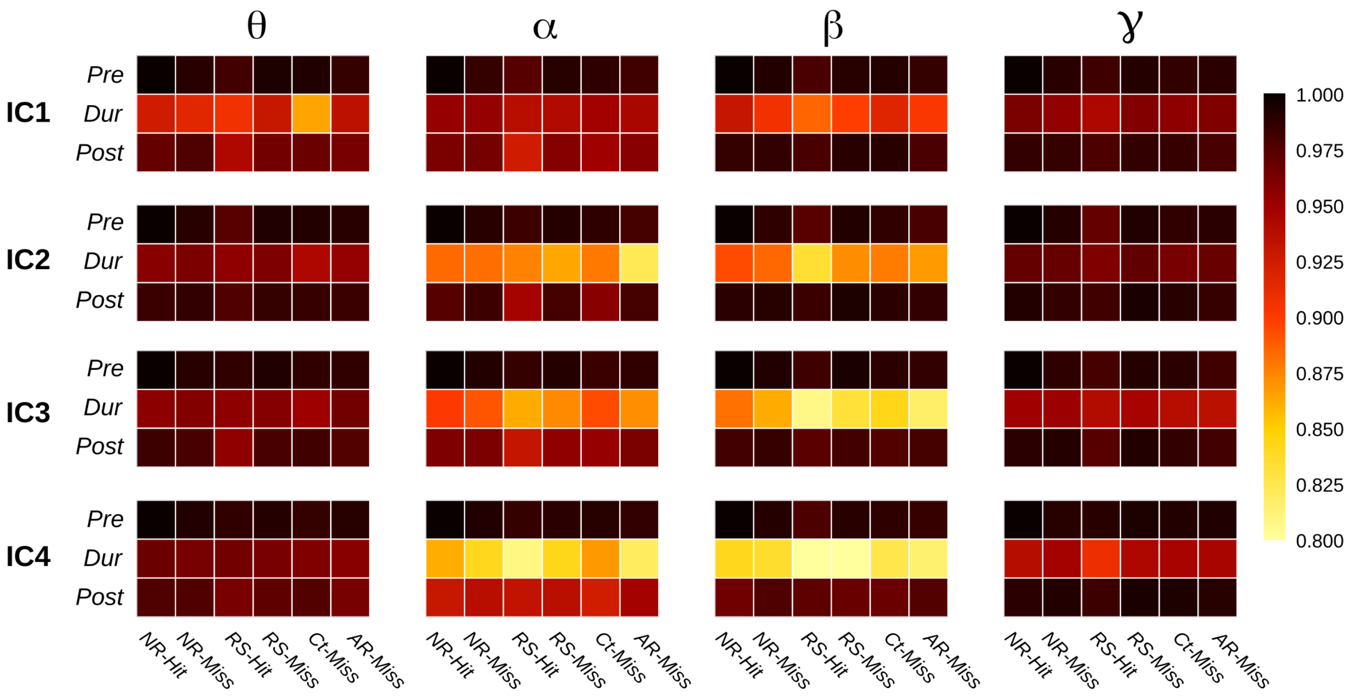

Figure S 1. The distributions of the single-trial oscillatory element largely overlapped across the different trial categories. Element $< \text{IC1, Pre}, \theta >$ (top left cell) serves here as a reference and the density range overlap is measured as the Bhattacharya distance between the distributions.

Table S 1. Break-up of outgoing connections from the movement error node to the oscillatory portraits.

Trial Category	Total number of outgoing connections (from Mvt. Error)	Spatial				Spectral				Temporal		
		IC1	IC2	IC3	IC4	θ	α	β	γ	Pre	During	Post
AR-Miss	2	50%	50%	-	-	50%	-	50%	-	-	50%	50%
Ct-Miss	2	-	50%	50%	-	100%	-	-	-	-	100%	-
NR-Hit	12	18.2%	36.4%	18.2%	27.3%	36.4%	27.3%	18.3%	18.2%	18.2%	72.7%	9.1%
RS-Hit	13	33.3%	16.7%	33.3%	16.7%	33.3%	25.0%	8.3%	33.3%	25%	50%	25%

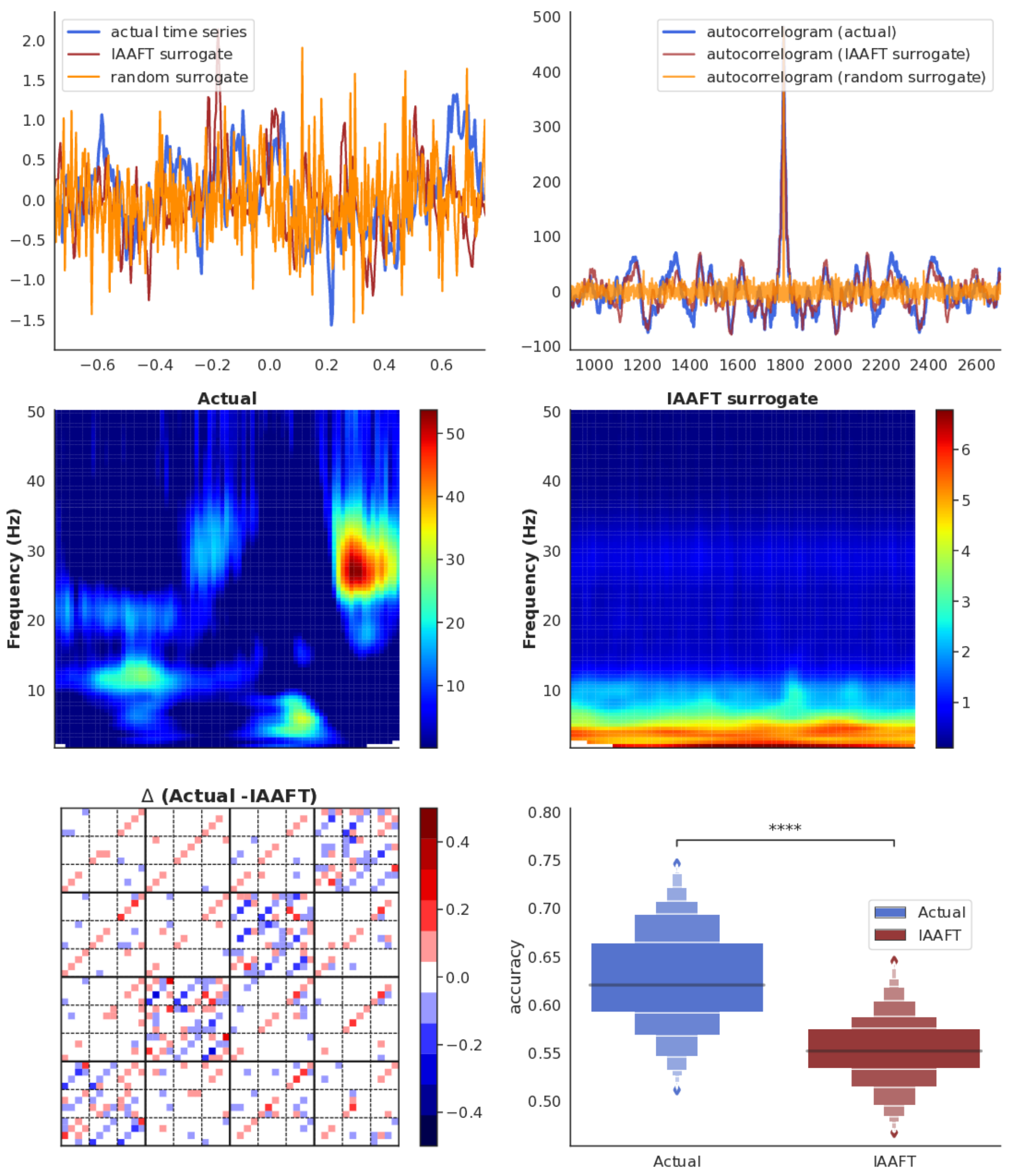

Figure S 2. Iteratively adjusted amplitude fourier transform (IAAFT) surrogates show a paucity of effective connectivity between the spatial components and show marginally better trial categorization than chance level. A) An example time series for a subject for IC1 shown in blue, it's IAAFT surrogate time series in maroon and its random surrogate time series in orange. B) The autocorrelogram for actual time series (blue), IAAFT surrogate (maroon) and random surrogate (orange). Note IAAFT surrogate conserves the autocorrelogram of the original signal as opposed to the random surrogate. C) Spectrogram for the example subject for IC1 averaged over all the trials. D) Spectrogram for the example subject for IC1 averaged over all the trials but for IAAFT surrogates of the original signal. E) Difference between the coefficients of the effective connectivity matrix of original signal (blue in A,B and spectrogram in C) and IAAFT surrogate signal (maroon in A,B and spectrogram in D). F) Accuracy of pairwise separation of trial categories for original and IAAFT surrogate signals. The IAAFT surrogate signals show significantly worse than actual signals(0.63 vs 0.55, two-sided sample t-test: 15.95, p=0.0), indicating that relationship between spatio-temporal-spectral components is essential to characterize the visuomotor processes.

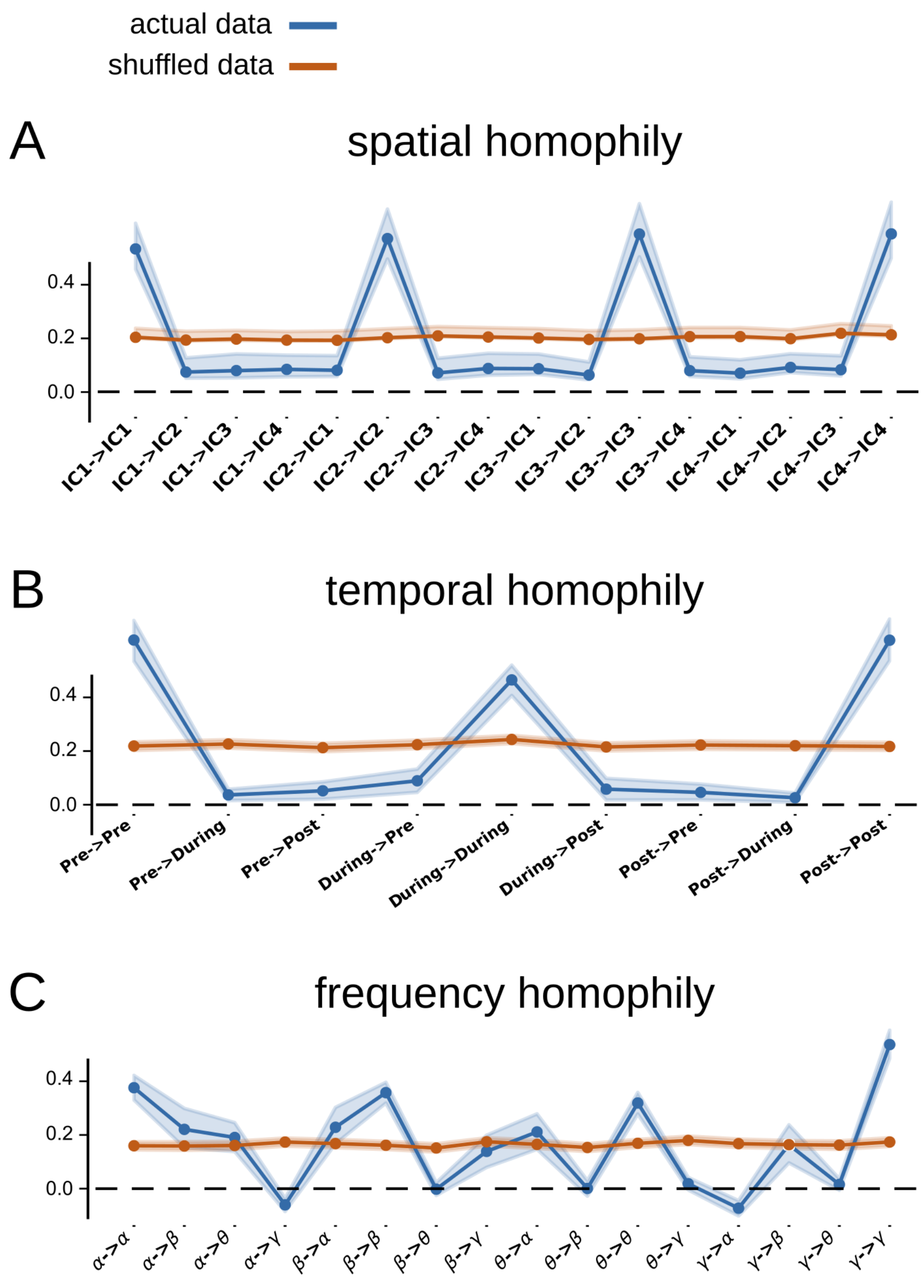

Figure S 3. The effective connectivity (EC) network was highly homophilic. A-C) Average weight in the spatial, temporal and frequency homophily ratios, respectively. The combinations of node pairs are indicated on the x-axis. The average weights are plotted for the actual effective connectivity networks (blue) and their shuffled versions (orange). All the networks for all trials categories and subjects were pooled together. The spatial node pairs belonging to the same brain area (e.g IC1 $\leftrightarrow$ IC1) show increased average weight, which indicates that stronger effective connectivity between same spatial nodes irrespective of their temporal and spectral features. The shuffled version of the network shows no pattern with respect to spatial node pairs. We quantify the total degree of homophily as the ratio between the average of homophilic weights (i.e. the weights of links between nodes with same values of the considered label type) and the total weight. The degree of spatial homophily is thus evaluated to 73%. B and C) Same as A) but for temporal (85%) and spectral (62%) homophily rations, respectively.

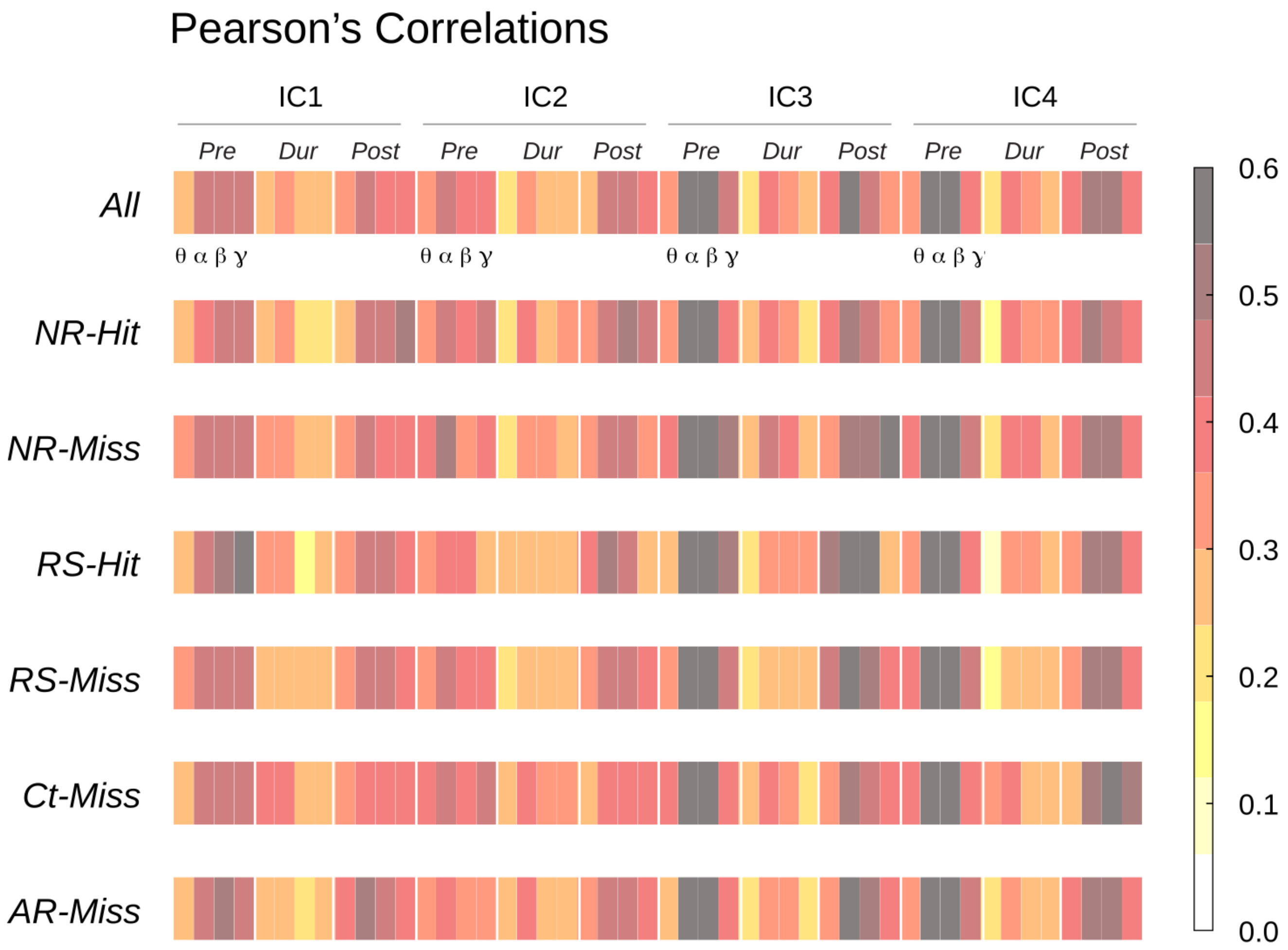

Figure S 4. To generate effective connectivity (EC) networks, single-trial values of each individual oscillatory element were predicted from the values of the others. For each oscillatory element, the prediction performance was measured using Pearson's correlation between the actual and the predicted values. This was performed for all the trial categories confounded (top row), as well as for the different trial categories separately.

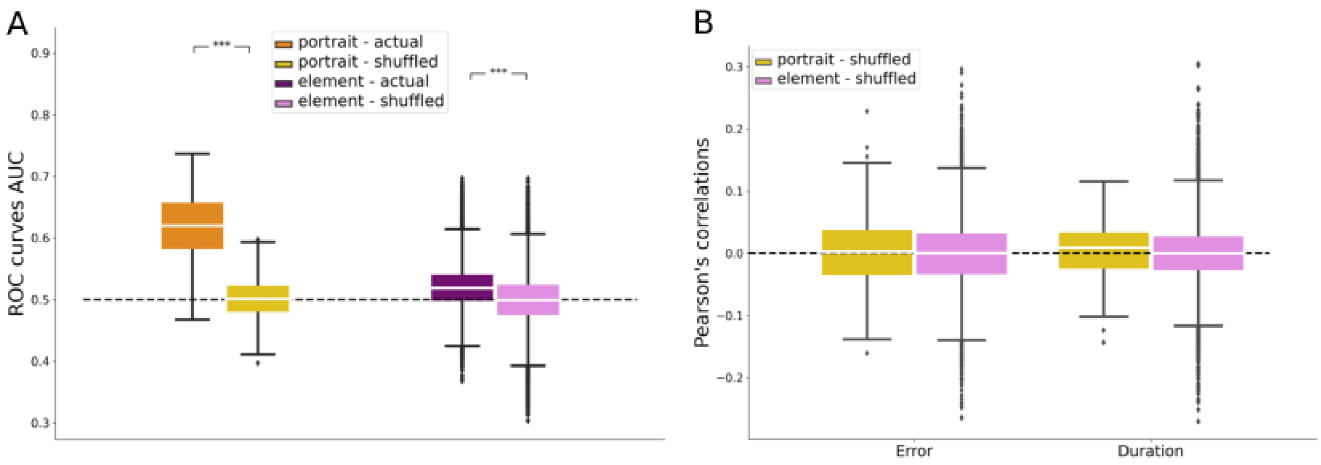

Figure S 5. Higher predictive power of oscillatory portraits over elements is not due to increased number of predictor variables. A) The accuracy of pairwise separation of trial categories measured as an area under the curve for the ROCs was calculated for oscillatory portraits and elements. In the shuffled versions of both elements and portraits, the trial categories in the training set were shuffled, so that the structure and number of predictor variables is conserved but the relationship between the trial categories and predicting mean power is disrupted. The shuffled models are unable to predict the trial categories and show chance level accuracy for both elements (0.52 vs 0.5, two-sided sample t-test: 60, p=0.0) and portraits (0.63 vs 0.5, two-sided sample t-test: 50.5, p=0.0). B) The Pearson's correlations between the actual and predicted movement error and duration is centered at 0 for the shuffled mode, showing no predictability and independence of the number of predictor variables.

Table S 2. Break-up of incoming connections to the movement duration node from the oscillatory portraits.

Trial Category	Total number of outgoing connections (to Mvt. Duration)	Spatial				Spectral				Temporal		
		IC1	IC2	IC3	IC4	θ	α	β	γ	Pre	During	Post
AR-Miss	6	50%	-	33.3%	16.7%	50%	-	33.3%	16.7%	66.7%	-	33.3%
Ct-Miss	3	33.3%	-	66.7%	-	33.3%	-	66.7%	-	-	33.3%	66.7%
NR-Hit	4	33.3%	-	66.7%	-	-	66.7%	33.3%	-	66.7%	-	33.3%
NR-Miss	3	100%	-	-	-	33.3%	-	33.3%	33.3%	33.3%	33.3%	33.3%
RS-Hit	10	22.2%	22.2%	33.3%	22.2%	-	11.1%	44.4%	44.4%	44.4%	11.1%	44.4%

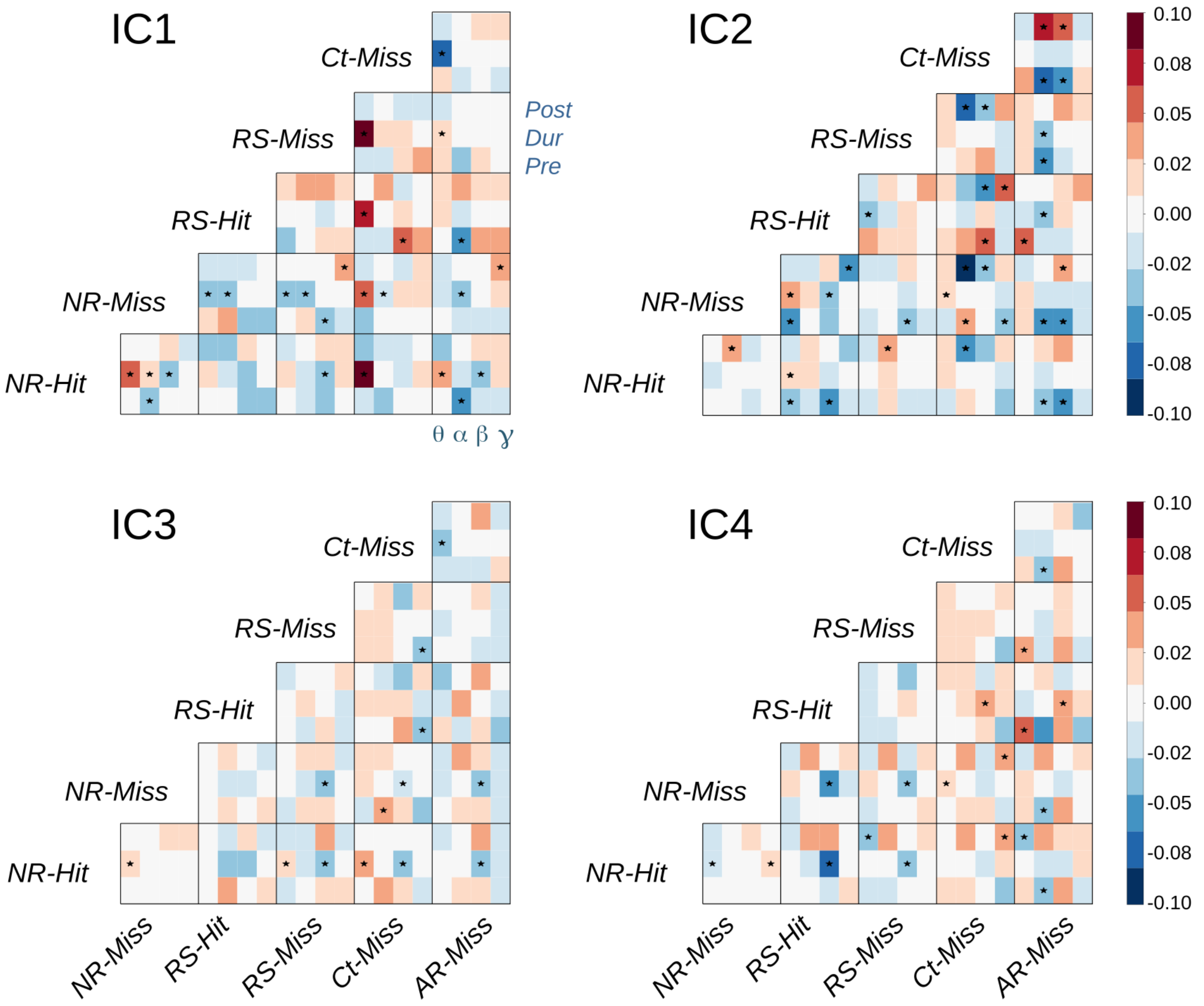

30 **Figure S 6.** Coefficients of each oscillatory element for pairwise separation between the trial categories. The coefficients can take positive/negative values
31 as indicated by the colors (warm = positive, cool = negative). Significant ($p < 0.05$) coefficients are marked with '*'. Negative(positive) values indicate that the
32 considered element has smaller(larger) power for trials of the category in the row than for trials of the category in the column.

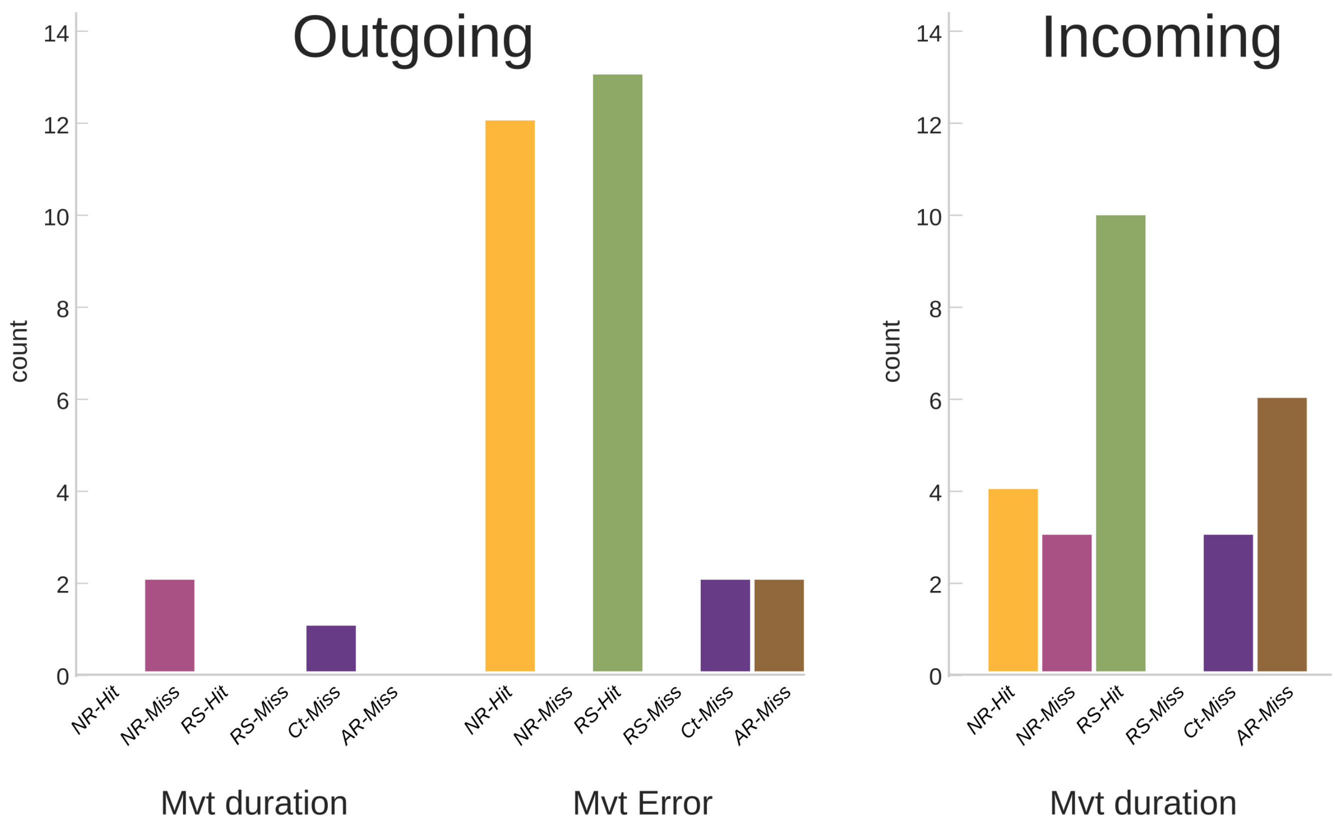

Figure S 7. A) Number of outgoing connections from Movement Duration and Movement Error nodes in a thresholded network (only connections stronger than 94 percentile of weight distribution pooled over all trial categories were considered). Movement error, especially RS-Hit and NR-Hit have high number of outgoing connections. B) Movement error in any trial category had any strong incoming projections. Whereas Movement duration nodes have high number of incoming projections, especially for RS-Hit and AR-Miss. The breakdown of these connections are listed in Table S 1 and S 2
